# Vitamin D levels and bone mineral density of middle-aged premenopausal female football and volleyball players in Japan: a cross-sectional study

**DOI:** 10.1186/s13102-024-00938-x

**Published:** 2024-07-02

**Authors:** Kimiko Sakamoto, Takayuki Miyamori, Yuki Someya, Masashi Nagao, Yoshihiko Ishihara, Yohei Kobayashi, Yu Shimasaki, Junko Imai, Takeshi Ono, Hiroshi Ikeda, Kohzo Tashima, Masafumi Yoshimura

**Affiliations:** 1 Graduate School of Health and Sports Science, Juntendo University, Chiba, Japan; 2 Department of Physical Therapy, Faculty of Health Science, Juntendo University, Tokyo, Japan; 3 Graduate School of Health Science, Juntendo University, Tokyo, Japan; 4 Graduate School of Health and Sports Science, Juntendo University, Chiba, Japan; 5 Department of Orthopedic Surgery, Faculty of Medicine, Juntendo University, Tokyo, Japan; 6 Medical Technology Innovation Center, Juntendo University, Tokyo, Japan; 7 School of Science and Technology for Future Life, Tokyo Denki University, Tokyo, Japan; 8 J Medical Oyumino, Chiba, Japan; 9 Department of Sports Science, Faculty of Health and Sports Science, Juntendo University, Chiba, Japan; 10 Japan Football Association, Tokyo, Japan

**Keywords:** Bone mineral density, 25-hydroxy vitamin D, Middle-aged, Premenopausal, Football, Volleyball, Exercise habits

## Abstract

**Background:**

The number of football teams in senior categories has increased. As outdoor sports entail players being exposed to sunlight, playing football may contribute to maintaining vitamin D stores and body mineral density while preventing osteoporosis. This study aimed to determine the bone mineral density and vitamin D levels in middle-aged premenopausal female football players.

**Methods:**

Participants were premenopausal females in their 40s. We evaluated bone mineral density of the second to the fourth lumbar vertebrae and femoral neck, serum 25-hydroxy vitamin D (25-OHD) levels, which is an indicator of vitamin D stores, and body composition. In addition, we administered a questionnaire survey on exercise habits and lifestyle. Ninety-two participants were categorised into three groups: the football group (*n* = 27), volleyball group (*n* = 40), and non-exercise group (*n* = 25).

**Results:**

Bone mineral density was higher in the football and volleyball groups than in the non-exercise group (*P* < 0.01). The volleyball group had a significantly higher bone mineral density of the lumbar spine and femoral neck than the non-exercise group (*P* < 0.01). The football group had a significantly higher bone mineral density of the femoral neck than the non-exercise group (*P* < 0.01). Although the football group had played fewer years than the volleyball group (*P* < 0.01), serum 25-OHD levels were the highest in the football group and were significantly higher than those in the volleyball and non-exercise groups (*P* < 0.01).

**Conclusions:**

Middle-aged premenopausal football players had higher body vitamin D levels and bone mineral densities than non-active females. These results suggest that playing football may contribute to the prevention of osteoporosis.

**Trial registration:**

UMIN Clinical Trials Registry UMIN000054235. 2024/04/23. Retrospectively registered.

**Supplementary Information:**

The online version contains supplementary material available at 10.1186/s13102-024-00938-x.

## Background

Football is one of the world’s most popular sports, with approximately 300 million players globally [1]. In Japan, the number of football players over 40 years old has been increasing [2] especially in male and female senior football [3].

Playing football has several health benefits [4–6]. Social networks are built through football, which improves health and promotes long-term physical activity [4]. Additionally, females and older males who play small-sided football games have significantly lower blood pressure, lower resting heart rate, and increased muscle mass and bone density [5, 6]. Moreover, older males who continuously play football have a significantly higher whole-body bone mineral density (BMD) and BMD of the proximal femur than males of the same ages who do not perform daily exercises [7]. It has also been reported that playing football increases the BMD of the femoral shaft and trochanter in sedentary middle-aged premenopausal females [8]. Although several health benefits of playing football have been reported, few studies have focused on the bone health of females who play football [7–10]. Bone health is particularly problematic for females aged ≥ 40 years due to hormonal imbalances [11].

Osteoporosis is a disease wherein bone strength becomes fragile and the risk of fracture increases due to reduced bone density and deterioration of bone quality [12, 13]. Among the risk factors of osteoporosis, modifiable factors include lack of exercise, vitamin D deficiency, inadequate calcium intake, and insufficient exposure to sunlight [12, 13]. There is a significant correlation between blood vitamin D levels and BMD in females aged 9–25 years and postmenopausal women aged 55–74 years [14–16]; therefore, vitamin D is considered an important factor in bone metabolism [17]. Vitamin D is produced in the skin through ultraviolet light and is significantly and positively associated with sunlight exposure [17, 18]. A study investigating seasonal variations in blood vitamin D levels in female university athletes reported that the football players who play outdoors had significantly higher blood vitamin D levels throughout the year compared to basketball and volleyball players who play indoors [19]. Hence, the implementation of outdoor physical activity and exposure to sunlight is necessary to prevent osteoporosis and BMD loss [12, 13]. Football is one of the most common outdoor sports; however, to date, the BMD and blood vitamin D levels in premenopausal middle-aged female football players remain unknown. Clarification of these levels may provide basic data for verifying whether playing football regularly can contribute to the prevention of BMD loss and osteoporosis.

Therefore, this study aimed to determine and compare blood vitamin D levels and BMD between premenopausal middle-aged females playing football and volleyball, which is a popular and competitive sport for this generation in Japan [20], and non-exercise groups.

We hypothesised that as football is an outdoor sport, the female football group will have higher blood vitamin D levels than the other groups. In addition, the female football and volleyball groups would have higher BMDs than the non-exercise group.

## Methods

### Participants and study design

This was a cross-sectional study, and participants were women aged 40–49 years. The football group consisted of football players who played outdoors. The volleyball group consisted of volleyball players who played indoors, and the non-exercise group consisted of women who did not exercise regularly (exercise < once a week). In this study, the football players had been playing in the Chiba Prefecture Female’s Football League for more than 2 years. The volleyball players had been playing in the Chiba Prefecture Senior Volleyball League for more than 2 years. Both groups had not participated regularly in any outdoor team sports for the past 2 years. The exclusion criteria were as follows: age < 40 years or > 50 years at the time of measurement, menopause (spontaneous or secondary to surgery), and consumption of drugs such as heparin, warfarin, cyclosporine, glucocorticoids, medroxyprogesterone, acetate, cancer drugs, and thyroid hormones.

**Fig. 1:**
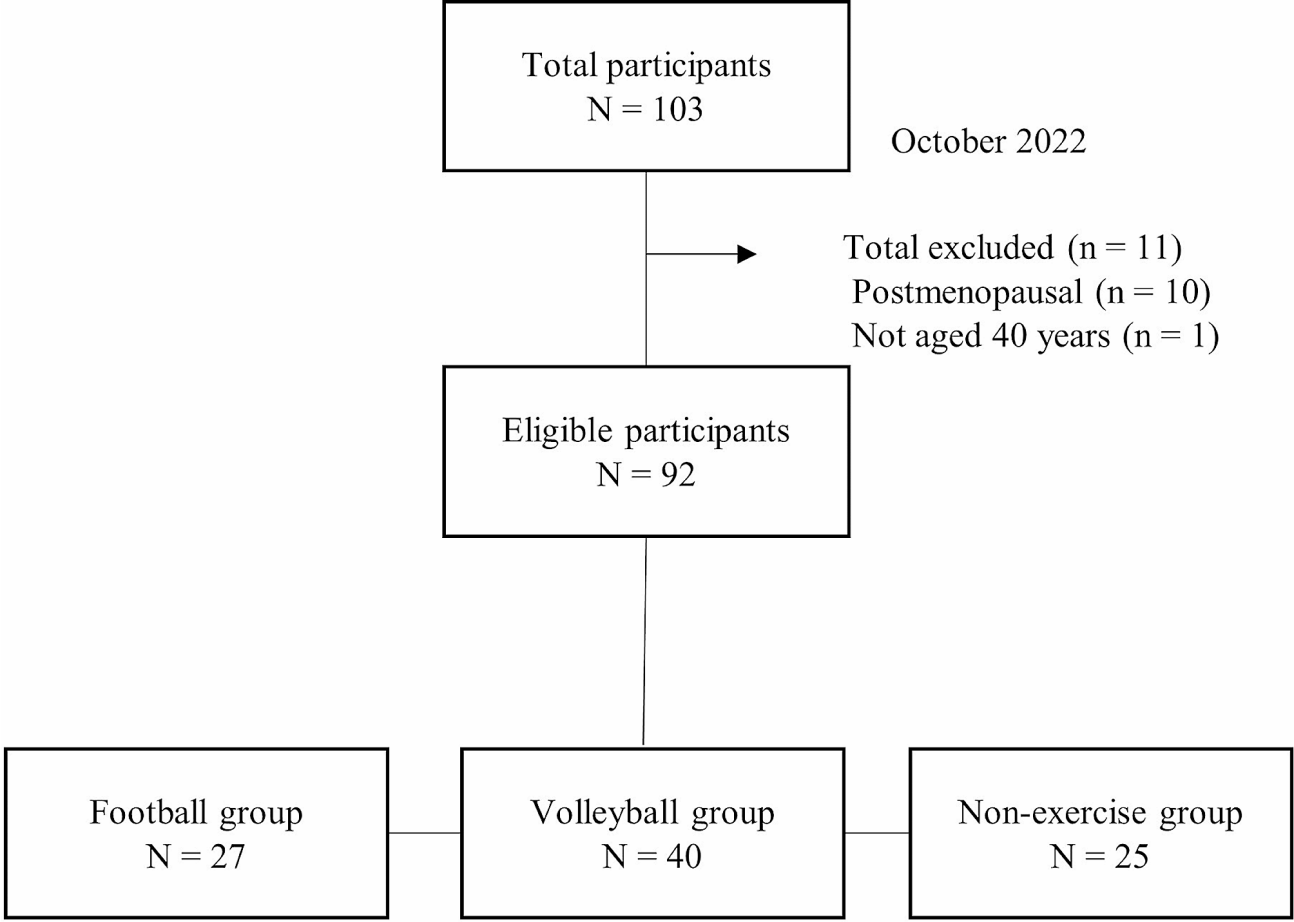
Participant selection flow chart

To recruit participants, posters were created regarding the study participation, and an invitation was sent by e-mail to the football and volleyball team members active in Chiba Prefecture in Japan. Those who were insufficiently active were invited to participate by directly handing posters to them. A total of 103 participants consented to participate in the study and in all measurements. Of these, 92 participants were divided into three groups for analysis: 27 in the football group, 40 in the volleyball group, and 25 in the non-exercise group. Ten postmenopausal women and one out-of-target age participant were excluded (Fig. 1). Measurements were conducted in October 2022 at Sakura Campus of Juntendo University. All measurements were obtained on the same day for the same participants.

The study was conducted with the approval of the Research Ethics Committee of the Faculty of Sports and Health Science of Juntendo University (Juntendo University Ethics Committee No. 2021 − 136) and in accordance with the Declaration of Helsinki. Participants were informed about the study in advance, and their informed written consent was obtained.

### Measurements and survey items

In this study, height, body composition, BMD, and blood vitamin D concentrations were measured, and a questionnaire survey related to lifestyle and exercise habits was administered.

#### Body composition measurements

Body composition was measured by bioelectrical impedance analysis (BIA) using InBody 730 (InBody Japan Inc., Tokyo, Japan) to determine body weight, skeletal muscle mass, body fat mass, body mass index (BMI, kg/m²), body fat percentage (%BF), and waist-to-hip ratio. The fat-free mass index (FFMI) was calculated based on %BF measured by the BIA method. The conditions for body composition measurements were no food intake within the last ≥ 8 h and no exercise prior to measurement.

#### Questionnaire survey

A questionnaire survey was conducted using an online form that included information on athletic history, nutritional intake, smoking status, drinking status, menstrual cycle, and previous childbirth.

For the athletic history of the volleyball and football groups, the survey asked about years of play, frequency of play per week, and playing hours per day. The number of years for continuous playing was based on the time of measurement; for interruptions of play, the interruption period was excluded.

The calcium intake score was calculated based on the calcium intake score reported by Ishii and Mizutani [21] in addition to the frequency of fish and mushroom consumption and the presence or absence of supplement intake within 1 week.

Smoking status was determined by examining whether there was a current smoking habit, a history of smoking in the past, or other smokers around the participants.

Drinking status was investigated regarding current alcohol consumption, frequency of alcohol consumption within 1 week, and amount of alcohol consumed per day.

Menstrual status was determined based on current menstruation, past menstrual irregularities, and age at which irregular menstruation occurred.

Regarding childbirth experience, the presence or absence of childbirth and the number of deliveries were obtained.

#### Measurement of BMD

BMD was measured by dual-energy X-ray absorptiometry using an X-ray bone densitometer (Hologic Discovery-A; Hologic Inc., Bedford, MA, USA). In this study, to diagnose osteoporosis, measurements were obtained at the following two sites: the second to the fourth lumbar vertebrae and the right femoral neck. The Z score for BMD, including same-age comparisons, was obtained, with the mean value assessed as being out of the age equivalent if < − 2.0 [22].

All BMD measurements were performed by an orthopaedic doctor and assessed according to the International Society for Clinical Densitometry protocol [23]. Calibration was performed before measurements using the phantom.

#### Blood vitamin D level measurements

Vitamin D stores in the body were assessed by measuring the serum 25-OHD concentration. Blood samples were collected and analysed using the chemiluminescent enzyme immunoassay method at the SRL Clinical Laboratory (Tokyo, Japan).

All measurements were carried out in October 2022.

### Statistical analysis

Participants’ age, height, and body composition are expressed as mean ± standard deviation, whereas BMD and blood 25-OHD concentration are expressed as mean values and 95% confidence intervals (CIs) for the error range. For between-group comparisons of participant characteristics, the Shapiro–Wilk test was used to check for normality. One-way analysis of variance was performed for items for which normality was found, and Tukey’s test was used for subsequent multiple comparisons. For items where normality was not found, the Kruskal–Wallis test was performed, and the Bonferroni test was used for subsequent multiple comparisons.

Categorical variables in the questionnaire were calculated as percentages within groups for each question item, and Pearson’s χ-square test was used for between-group comparisons.

For between-group comparisons of blood 25-OHD concentration, lumbar spine BMD, and femoral neck BMD, analysis of covariance was performed using age and BMI as confounders. Bonferroni’s test was performed for subsequent multiple comparisons.

As sample size calculation was not conducted before recruitment, a post hoc power analysis was performed to confirm the probability of a type-II error. Effect size (η²) and power were calculated using G*power 3.1.9.7 software.

All statistical analyses were conducted using IBM SPSS Statistics version 22 (IBM Corp., Armonk, NY, USA), with a significance level of 5%.

## Results

### Participants’ characteristics

#### Age, height, and body composition

The group comparisons for age, height, and body composition are presented in Table 1. The football group was significantly older than the volleyball and non-exercise groups (*P* = 0.01). Skeletal muscle mass was significantly higher in the football and volleyball groups than in the non-exercise group (*P* = 0.02 and *P* = 0.01, respectively). Skeletal muscle percentage was significantly higher in the football group than in the non-exercise group (*P* = 0.01) (no significant difference with the volleyball group, *P* = 0.06). FFMI was significantly higher in the football and volleyball groups than in the non-exercise group (*P* = 0.01, *P* = 0.01). Furthermore, the waist-to-hip ratio was significantly lower in the football group than in the non-exercise and volleyball groups (*P* = 0.01). No significant differences were found between groups in terms of height, weight, body fat mass, BMI, or %BF.

**Table 1:** Comparison of age, height, and body composition between groups

	Non-exercise (*n* = 25)	Volleyball (*n* = 40)	Football (*n* = 27)	*P*-value ^b^	Effect size	Power
Age (years)	44.0 ± 2.3^a^	44.3 ± 2.8	46.0 ± 2.7**	0.01	0.10	0.79
Height (cm)	160.2 ± 5.8	161.1 ± 5.5	159.9 ± 4.8	0.61	0.01	0.13
Weight (kg)	57.1 ± 7.8	60.1 ± 9.1	57.6 ± 7.8	0.38	0.03	0.25
Skeletal muscle mass (kg)	21.1 ± 2.1	23.1 ± 2.9*	22.8 ± 2.5*	0.02	0.10	0.80
Body fat amount (kg)	18.0 ± 5.9	17.7 ± 6.9	16.0 ± 5.0	0.48	0.02	0.20
Body mass index (kg/m²)	22.3 ± 3.2	23.3 ± 4.2	22.5 ± 2.6	0.79	0.01	0.16
Body fat percentage (%)	30.9 ± 6.4	28.7 ± 7.0	27.2 ± 5.5	0.11	0.05	0.44
Skeletal muscle percentage (%)	37.1 ± 3.4	38.7 ± 3.8	39.7 ± 3.0*	0.01	0.07	0.64
Fat-free mass percentage (kg/m²)	15.3 ± 1.1	16.3 ± 1.7*	16.3 ± 1.2*	0.01	0.02	0.82
Waist-hip ratio	0.85 ± 0.05	0.85 ± 0.05	0.82 ± 0.04**	0.01	0.97	0.78

^a^ Average ± standard deviation

^b^ P: analysis of covariance

* *P* < 0.05: vs. non-exercise

** *P* < 0.05: vs. volleyball

### Questionnaire survey

*Athletic competition status*

Table 2 presents the years of play. Table 3 presents the frequency of play. Table 4 presents the hours played per session for the volleyball and football groups.

**Table 2:** Comparison of years of play in the exercise group

	Volleyball	Football	P-value ^d^
	(*n* = 40)	(*n* = 27)	
<3 years ^b^	0	5	0.01
	(0.0) ^a^	(18.5)	
3–5 years	3	0	
	(7.5)	(0.0)	
5–10 years	8	11	
	(20.0)	(40.7)	
11–15 years	11	6	
	(27.5)	(22.2)	
15–20 years	4	1	
	(10.0)	(3.7)	
> 20 years ^c^	14	4	
	(35.0)	(14.8)	

^a^ Number (%)

^b^ <3 years: less than 3 years

^c^ >20 years: More than 20 years

^d^ P: analysis of covariance

**Table 3:** Comparison of frequency of play in the exercise group

	Volleyball	Football	P-value ^b^
	(*n* = 40)	(*n* = 27)	
< 1 time/month ^c^	1	1	0.59
	(2.5) ^a^	(3.7)	
2 times/month	3	2	
	(7.3)	(7.4)	
3 times/month	0	1	
	(0.0)	(3.7)	
< 1 time/week	12	7	
	(30.0)	(25.9)	
2 times/week	14	11	
	(35.0)	(40.7)	
3 times/week	5	5	
	(12.5)	(18.5)	
4 times/week	3	0	
	(7.5)	(0.0)	
> 5 times/week ^d^	2	0	
	(5.0)	(0.0)	

^a^ Number (%)

^b^ P: analysis of covariance

^c^ <1 time/month: less than 1 time

^d^ >5 times/week: more than 5 times

**Table 4:** Comparison of playing hours per time in the exercise group

	Volleyball	Football	P-value ^b^
	(*n* = 40)	(*n* = 27)	
< 1–2 h ^c^	27	12	0.17
	(67.5) ^a^	(44.4)	
2–3 h	11	13	
	(27.5)	(48.1)	
3–4 h	2	2	
	(5.0)	(7.4)	

^a^ Number (%)

^b^ P: analysis of covariance

^c^ <1–2 h: less than 1 h to 2 h

The football group exhibited the highest percentage of respondents who had been playing for 5–10 years (40.7%), whereas the volleyball group had the highest percentage of respondents who had been playing for > 20 years (35.0%). There was a significant difference between the groups in years of play (*P* = 0.01), with the ‘less than 3 years’ option showing a difference in frequency based on an adjusted residual analysis. The largest proportion of both groups played ‘twice a week’. The football group had the highest proportion of respondents (48.1%) who played ‘less than 2–3 hours’, whereas the volleyball group had the highest proportion (67.5%) who played ‘less than 1–2 hours’.

#### Nutritional intake status

The frequency of fish and mushroom intake per week, calcium intake scores, and supplement intake are presented in Tables 5, 6 and 7, and 8. No significant differences were observed between the groups for any outcome.

**Table 5:** Comparison of frequency of fish consumption within a week between groups

	Non-exercise	Volleyball	Football	P-value ^b^
	(*n* = 25)	(*n* = 40)	(*n* = 27)	
1 time	15	22	13	0.79
	(60.0) ^a^	(55.0)	(48.1)	
2 times	6	12	9	
	(24.0)	(30.0)	(33.3)	
3 times	4	4	4	
	(16.0)	(10.0)	(14.8)	
4 times	0	1	0	
	(0.0)	(2.5)	(0.0)	
5 times	0	0	1	
	(0.0)	(0.0)	(3.7)	
6 times	0	0	0	
	(0.0)	(0.0)	(0.0)	
> 7 times ^c^	0	1	0	
	(0.0)	(2.5)	(0.0)	

^a^ Number (%)

^b^ P: analysis of covariance

^c^ >7 times: more than 7 times/week

**Table 6:** Comparison of frequency of mushroom consumption within a week between groups

	Non-exercise	Volleyball	Football	P-value ^b^
	(*n* = 25)	(*n* = 40)	(*n* = 27)	
1 time	6	9	14	0.23
	(24.0) ^a^	(22.5)	(51.9)	
2 times	8	16	3	
	(32.0)	(40.0)	(11.1)	
3 times	7	6	4	
	(28.0)	(15.0)	(14.8)	
4 times	2	4	2	
	(8.0)	(10.0)	(7.4)	
5 times	1	3	2	
	(4.0)	(7.5)	(7.4)	
6 times	1	1	0	
	(4.0)	(2.5)	(0.0)	
> 7 times ^c^	0	1	2	
	(0.0)	(2.5)	(7.4)	

^a^ Number (%)

^b^ P: analysis of covariance

^c^ >7 times: more than 7 times/week

**Table 7:** Comparison of calcium intake score between groups

	Non-exercise	Volleyball	Football	*P*-value ^b^
	(*n* = 25)	(*n* = 40)	(*n* = 27)	
Calcium intake score	5.3 ± 2.3^a^	5.3 ± 2.5	6.3 ± 2.4	0.74

^a^ Number ± standard deviation

^b^ P: analysis of covariance

**Table 8:** Comparison of supplement intake or not between groups

	Non-exercise	Volleyball	Football	P-value ^b^
	(*n* = 25)	(*n* = 40)	(*n* = 27)	
Yes	6	9	8	0.80
	(24.0) ^a^	(22.5)	(29.6)	
No	19	31	19	
	(76.0)	(77.5)	(70.4)	

^a^ Number (%)

^b^ P: analysis of covariance

#### Smoking status

Table 9 presents the current smoking status, and Table 10 shows the presence or absence of smokers around the participants. No significant differences were found between groups for either outcome.

**Table 9:** Current smoking status

	Non-exercise	Volleyball	Football	P-value ^b^
	(*n* = 25)	(*n* = 40)	(*n* = 27)	
None	21	25	22	0.14
	(84.0) ^a^	(62.5)	(81.5)	
Past experience	2	6	4	
	(8.0)	(15.0)	(14.8)	
Yes	2	9	1	
	(8.0)	(22.5)	(3.7)	

^a^ Number (%)

^b^ P: analysis of covariance

**Table 10:** Smokers around

	Non-exercise	Volleyball	Football	P-value ^b^
	(*n* = 25)	(*n* = 40)	(*n* = 27)	
None	17	19	16	0.43
	(68.0) ^a^	(47.5)	(59.3)	
Roommate	6	16	10	
	(24.0)	(40.0)	(37.0)	
Not a roommate/others	2	5	1	
	(8.0)	(12.5)	(3.7)	

^a^ Number (%)

^b^ P: analysis of covariance

#### Drinking status

The frequency and amount of alcohol consumed per day are listed in Tables 11 and 12, respectively. No significant differences were found in either the frequency or amount of alcohol consumed per day.

**Table 11:** Comparison of current frequency of alcohol consumption between groups

	Non-exercise	Volleyball	Football	P-value ^b^
	(*n* = 25)	(*n* = 40)	(*n* = 27)	
None	6	13	8	0.07
	(24.0) ^a^	(32.5)	(29.6)	
< 1 time/week	4	13	3	
	(16.0)	(32.5)	(11.1)	
1 time/week	3	1	6	
	(12.0)	(2.5)	(22.2)	
2 times/week	4	3	6	
	(16.0)	(7.5)	(22.2)	
3 times/week	1	1	1	
	(4.0)	(2.5)	(3.7)	
4 times/week	1	2	0	
	(4.0)	(5.0)	(0.0)	
5 times/week	0	2	2	
	(0.0)	(5.0)	(7.4)	
6 times/week	0	0	0	
	(0.0)	(0.0)	(0.0)	
Every day	6	7	1	
	(24.0)	(17.5)	(3.7)	

^a^ Number (%)

^b^ P: analysis of covariance

^c^ <1 time/week: less than 1 time/week

**Table 12:** Comparison of amount of alcohol consumption per day between groups

	Non-exercise	Volleyball	Football	P-value ^b^
	(*n* = 25)	(*n* = 40)	(*n* = 27)	
Non	6	14	10	0.54
	(24.0) ^a^	(35.0)	(37.0)	
< 20 g ^c^	11	17	12	
	(44.0)	(42.5)	(44.4)	
20–40 g	7	6	4	
	(28.0)	(15.0)	(14.8)	
40–60 g	0	0	1	
	(0.0)	(0.0)	(3.7)	
60–80 g	0	0	0	
	(0.0)	(0.0)	(0.0)	
> 80 g ^d^	1	3	0	
	(4.0)	(7.5)	(0.0)	

^a^ Number (%)

^b^ P: analysis of covariance

^c^ <20 g: less than 20 g/day

^d^ >80 g: more than 80 g/day

#### Menstrual status

Seventy-five participants (74.3%) answered ‘no’ to the question ‘have you ever experienced amenorrhoea or rare menstruation in the past, except before or after childbirth’. Of the 17 participants who had ‘ever’ experienced amenorrhoea, six experienced amenorrhoea or rare menstruation in their teenage years. No significant differences were observed between the groups in terms of previous amenorrhoea or amenorrhoea.

#### Childbirth experience

The number of births is presented in Table 13. The highest number of deliveries experienced was ‘twice’ in the football, volleyball, and non-exercise groups. There were no significant differences between groups in terms of the number of births.

**Table 13:** Comparison of number of childbirth experiences between groups

	Non-exercise	Volleyball	Football	P-value ^b^
	(*n* = 25)	(*n* = 40)	(*n* = 27)	
None	1	0	6	0.06
	(0.0) ^a^	(0.0)	(22.2)	
1 time	2	6	3	
	(8.0)	(15.0)	(11.1)	
2 times	17	21	11	
	(68.0)	(52.5)	(40.7)	
3 times	4	12	6	
	(16.0)	(30.0)	(22.2)	
> 4 times ^c^	1	1	1	
	(4.0)	(2.5)	(3.7)	

^a^ Number (%)

^b^ P: analysis of covariance

^c^ >4 times: more than 4 times

### Multivariate analysis of blood vitamin D concentration and BMD

The results of the multivariate analysis for blood 25-OHD concentrations and BMD, with age and BMI as adjustment factors, are presented in Table 14. Blood 25-OHD concentrations were higher in the football group than in the non-exercise and volleyball groups (*P* < 0.01, η² = 0.15). Furthermore, lumbar spine BMD was higher in the volleyball group than in the non-exercise group (*P* < 0.01, η² = 0.16), and femoral neck BMD was higher in the football and volleyball groups than in the non-exercise group (*P* < 0.01, η² = 0.23). Z-scores for lumbar spine and femoral neck BMD were higher in the football and volleyball groups than in the non-exercise group (*P* = 0.03 and *P* < 0.01, η² = 0.16, respectively).

**Table 14:** Multivariate analysis of 25-OHD levels and bone mineral density between groups

	Non-exercise	Volleyball	Football	P-value ^b^	Effect size	Power
	(*n* = 25)	(*n* = 40)	(*n* = 27)			
25-OHD (ng/mL)	14.9	15	19.4**	< 0.01	0.15	0.95
	(12.7–17.0) ^a^	(13.3–16.7)	(17.7–21.1)			
Bone density of lumbar spine (g/cm²)	1.024	1.151*	1.104	< 0.01	0.16	0.96
	(0.970–1.077)	(1.113–1.189)	(1.056–1.152)			
Bone density of femoral neck (g/cm²)	0.708	0.824*	0.833*	< 0.01	0.23	0.99
	(0.664–0.751)	(0.791–0.858)	(0.797–0.870)			
Bone density of Lumbar spine (Z score)	0.140	1.108*	0.811*	< 0.01	0.16	0.97
	(-0.253–0.533)	(0.823–1.392)	(0.450–1.172)			
Bone density of femoral neck (Z score)	-0.352	0.777*	0.948*	< 0.01	0.24	0.99
	(− 0.764–0.060)	(0.451–1.104)	(0.582–1.314)			

^a^ Average (95% confidential interval)

^*b*^*P*: analysis of covariance

^c^ Confounders: age, body mass index (BMI, only BMI for Z score)

*P < 0.05: vs. non-exercise; **P < 0.05: vs. volleyball

25-OHD, 25-hydroxy vitamin D

## Discussion

This study examined BMD and blood vitamin D levels of premenopausal middle-aged female football players and compared them with those of females of the same age who played volleyball and did not exercise regularly. The results showed that the football and volleyball groups had significantly higher Z-scores for the femoral neck and lumbar spine BMD than the non-exercise group. Furthermore, blood vitamin D levels in the football group were significantly higher than those in the volleyball and non-exercise groups.

### Blood vitamin D levels

The football group had the highest blood vitamin D levels compared with the other two groups, whereas the volleyball and non-exercise groups had comparable values. Blood vitamin D levels are increased by the production of vitamin D in the skin due to ultraviolet radiation associated with skin exposure to the sun [24]. Therefore, because those players in the football group regularly played in summer (July to September 2022), it is possible that body vitamin D stores may have increased during football matches; these are outdoor activities that provide more frequent opportunities for exposure to sunlight. Maruyama et al. [19] reported that blood 25-OHD levels were higher throughout the year in outdoor sports athletes than in indoor sports athletes. In addition, a study investigating the relationship between sunlight exposure and blood 25-OHD concentrations in adult males living in urban India found that blood 25-OHD concentrations increased with increasing sunlight exposure [25]. Furthermore, Ono et al. [24] investigated seasonal variations in blood 25-OHD concentrations of 197 members of the general population aged 20–68 years living in the Tokai region of Japan and reported that the mean values were lowest during late winter and highest during late summer. Compared to the average annual sunshine hours in the Tokai region, the results showed that vitamin D production in the skin due to exposure to sunlight had a significant effect on blood 25-OHD concentrations [24]. Therefore, in line with these previous studies, we considered that the football group, whose members played outdoors, had significantly higher blood 25-OHD concentrations than the other two groups, whereas those of the volleyball group, whose members played indoors, were comparable to those of the non-exercise group.

However, the cut-off value for a satisfactory blood 25-OHD concentration is 20 ng/mL or higher, although neither group was in a satisfactory state in the present study. As blood 25-OHD levels fluctuate, reflecting body production from sun exposure and food intake [17], it is possible that the participants in this study did not frequently consume vitamin D-rich foods. Vitamin D is abundant in fish and mushrooms [26]. Regarding this study’s questionnaire, most of the respondents stated their frequency of consumption of those foods as once/week. Owing to this low vitamin D intake, participants in this study did not have vitamin D levels above the cut-off value, even in the football group.

### BMD

The football and volleyball groups exhibited significantly higher Z-scores for femoral neck and lumbar spine BMD than the non-exercise group.

Volleyball is a sport that combines intermittent active and passive play and is based on the repetition of movements such as vertical jumps, spikes, blocks, and serves [27]. In a study by Alfredson et al. [28] that compared the BMD in 13 female volleyball players and 13 females who did not regularly exercise, the volleyball players had a significantly higher BMD in the lumbar spine, femoral neck, femoral greater trochanter, and femur on the non-dominant leg side than non-exercising females. Ito et al. [29] studied the BMD of pre-, peri-, and postmenopausal females who habitually played volleyball and found a significantly higher BMD in the lumbar spine, tibia, and calcaneus in the volleyball group than in the non-exercise group. These previous studies suggest that repetitive jumping movements characteristic of volleyball may provide a vertical mechanical stimulus to the bones and promote bone formation, resulting in higher lumbar spine and femoral neck BMD.

Football is characterised by repetitive running with quick changes in direction, acceleration and deceleration, jumping, and kicking [30]. Alfredson et al. [31] measured and compared the BMD in 16 female football players and 13 females who did not exercise regularly. They reported that female footballers had significantly higher BMD in the lumbar spine and femoral neck than the non-exercising females. In addition, Hage [32] investigated the effect of football practice on hip BMD in females and reported that the BMD at the femoral neck was significantly higher in the football group than in the non-exercise group. Additionally, other measures of bone strength, such as bone cross-sectional area and cortical thickness, were also significantly higher in the female football group. Hence, continued competition-specific movements in football may increase femoral neck BMD, particularly due to mechanical stimulation of the femoral neck, which constitutes the hip joint.

In addition to skeletal muscle mass, skeletal muscle percentage, which is the ratio of skeletal muscle mass to body weight, and FFMI were significantly higher in the football group than in the non-exercise group, while skeletal muscle mass and FFMI were significantly higher in the volleyball group than in the non-exercise group. The skeletal and muscular systems are considered interdependent, and a large cross-sectional study in the United States of America reported a positive correlation between skeletal muscle mass and whole-body, lumbar spine, and pelvic BMD [33, 34]. Therefore, it was likely that the BMD would be higher in the football and volleyball groups, which had a higher skeletal muscle mass, skeletal muscle percentage, and FFMI.

### Limitations

This study had several limitations. As this study had a cross-sectional design, it was not possible to explain the causal relationship between the playing of football and blood vitamin D levels and BMD. BMD reaches its maximum value in the late teenage years [35]; therefore, it is important to know whether regular physical activity was a habit during adolescence. However, the questionnaire administered in the present study did not provide details on the regular exercise habits of the participants during their teenage years. In addition, as vitamin D is produced by UV radiation to the skin, it is possible that the use of sunscreen creams may have affected vitamin D production in the participants. Therefore, future research should investigate the association between sunscreen cream use and participants’ blood vitamin D levels. Furthermore, the amount of UV radiation required to produce vitamin D in the skin is influenced by season and latitude [18], and the amount of vitamin D produced by exposure to sunlight varies according to skin colour [36]. This study did not evaluate the amount of sun exposure, and the participants were Japanese females only. Therefore, the results of the present study need to be examined in a larger sample size before they can be generalised. Finally, the present study did not include bone formation makers, such as osteocalcin, and bone resorption markers, such as amino-terminal cross-linked telopeptide of type 1 collagen, for data validation.

## Conclusions

Premenopausal female football players in their 40s had a significantly higher femoral neck BMD and blood vitamin D concentrations than the non-exercise female group. Although football players had fewer years of play than volleyball players, their blood vitamin D levels were significantly higher than those in the other groups. As blood vitamin D levels are associated with increased bone metabolism and BMD, continuously playing football may contribute to the prevention of future postmenopausal bone loss. Future studies should prospectively consider the influence of detailed confounders such as training hours, nutritional intake, and amount of sun exposure between the groups.

### Electronic supplementary material

Below is the link to the electronic supplementary material.

Supplementary Material 1

## Data Availability

Raw data were generated at the Faculty of Health Science, Juntendo University. Derived data supporting the findings of this study are available from the corresponding author, Takayuki Miyamori, on request.

## Abbreviations

25-OHD: 25-hydroxy Vitamin D
BIA: Bioelectrical Impedance Analysis
BMD: Bone Mineral Density
BMI: Body Mass Index
CI: Confidence Interval
FFMI: Fat-Free Mass Index
