# Supplementary material for: Vitamin D levels and bone mineral density of middle-aged premenopausal female football and volleyball players in Japan: a cross-sectional study
Source: BMC Sports Sci Med Rehabil. 2024 Jul 2;16:147. doi: 10.1186/s13102-024-00938-x (PMC11221148; doi:10.1186/s13102-024-00938-x)
Supplement: Supplementary file 1 — Supplementary Material 1 [file 13102_2024_938_MOESM1_ESM.docx]

**Additional file 1. Questionnaire on exercise habits and lifestyle**

**Basic information**

| - Please enter your name |  |
| --- | --- |
| - Please select your gender | ・Female  ・Other |
| - How old are you? | 1．Less than 39 years old  2．40 years old  3．41 years old  4．42 years old  5．43 years old  6．44 years old  7．45 years old  8．46 years old  9．47 years old  10．48 years old  11．49 years old  12．More than 50 years old |
| - What is your height? |  |
| - What is your body weight? |  |

**１．Athlete history**

| 1-1．Do you play volleyball or football? | ・Football  ・Volleyball  ・Neither |
| --- | --- |

For those who selected “volleyball or football”

| 1-1-1．How many years have you been playing so far?  *If you stopped playing for any considerable period, please select the number of years excluding that period. | 1．Less than 3 years  2．3–5 years  3．5–10 years  4．11–15 years  5．15–20 years  6．More than 20 years |
| --- | --- |
| 1-1-2．What is your average frequency of playing volleyball or football? | 1．Less than 1 time / month  2．1 time / month  3．2 times / month  4．3 times / month  5．1 time / week  6．2 times / week  7．3 times / week  8．4 times / week  9．5 times / week  10．6 times / week  11．7 times / week |
| 1-1-3．What is the average number of hours you play volleyball or football? | 1．Less than 1 hour  2．1–2 hours  3．2–3 hours  4．3–4 hours  5．More than 4 hours |

**2．Current exercise habits**

＊Exercise is defined as physical exercise for the purpose of body fitness

＊Please answer if you answered「Neither」in 1-1 above

| 2-1．Do you currently have an exercise habit? | 1．No  2．Less than 1 time / week  3．1 time / week  4．2 times / week  5．3 times / week  6．4 times / week  7．5 times / week  8．6 times / week  9．7 times / week |
| --- | --- |
| 2-2．What is the average period of exercise? | 1．Less than 15 minutes  2．15–30 minutes  3．30–45 minutes  4．45–60 minutes  5．More than 60 minutes |
| 2-3．What is the main exercise setting? | ・Indoor  ・Outdoor |

**3．Diet　【Calcium intake status】　Ishi & Mizutani 2018**

| 3-1．How often do you drink milk?  (Please answer taking the amount per serving as 1 glass ⁓ 160㎖) | 1．Hardly drink  2．1–2 times / month  3．1–2 times / week  4．3–4 times / week  5．Almost every day |
| --- | --- |
| 3-2．How often do you eat yoghurt?  (Please answer taking the amount per serving as 100 g) | 1．Hardly eat  2．1–2 times  3．3–4 times  4．Almost every day  5．Two almost every day |
| 3-3．Do you often consume other dairy products (cheese, skimmed milk, etc.)?  Please answer as 1 slice of cheese (20 g) and 1.5 tbsp (10 g) of skim milk per serving | 1．Hardly eat  2．1–2 times / week  3．3–4 times / week  4．Almost every day  5．Two or more types every day |
| 3-4．Do you often eat soya, natto, or other legumes?  Please answer taking 1 pack of natto (fermented soybeans), 1 small bowl of cooked soybeans, or 2 tablespoons of soybean flour as 1 serving. | 1．Hardly eat  2．1–2 times / week  3．3–4 times / week  4．Almost every day  5．Two or more types every day |
| 3-5．How often do you eat soya products such as tofu and thick fried tofu?  Please answer the taking the amount of serving as 1/4 tofu or 1 thick fried bean curd. | 1．Hardly eat  2．1–2 times / week  3．3–4 times / week  4．Almost every day  5．Two or more types every day |
| 3-6．Do you often eat green vegetables such as spinach?  (Please answer taking the amount per serving to be one small bowl of soaked greens) | 1．Hardly eat  2．1–2 times / week  3．3–4 times / week  4．Almost every day  5．Two or more types every day |
| 3-7．How often do you eat seaweed? | 1．Hardly eat  2．1–2 times / week  3．3–4 times / week  4．Almost every day  5．Two or more types every day |

**3．Diet　【Regarding intake of other foods】**

| 3-8．How often do you eat fish? | ・1 time / week  ・2 times / week  ・3 times / week  ・4 times / week  ・5 times / week  ・6 times / week  ・More than 7 times / week |
| --- | --- |
| 3-9．How often do you eat mushrooms? | ・1 time / week  ・2 times / week  ・3 times / week  ・4 times / week  ・5 times / week  ・6 times / week  ・More than 7 times / week |

**4．Smoking　【Current smoking status】**

| 4-1．Are you currently smoking? | ・No  ・No, but smoked in the past  ・Yes |
| --- | --- |

**4．Smoking　【Passive smoking status】**

| 4-2．Are there current smokers around you? | ・None  ・Roommate  ・Not a roommate/others |
| --- | --- |

**5．Drinking　【Regarding current drinking status】**

＊For the item on the amount of alcohol consumed, please answer the guideline for 20 g of pure alcohol as follows: beer 500㎖ / sake 1 g / shochu 0.6 g / whisky 60㎖ / wine 1 glass / canned shochu 500㎖.

| 5-1．Do you currently drink alcohol? | 1．No  2．1 time / week (social consumption)  3．1 time / week  4．2 times / week  5．3 times / week  6．4 times / week  7．5 times / week  8．6 times / week  9．Everyday |
| --- | --- |

**5．Drinking　【Regarding the current amount of alcohol consumed】**

＊For the item on the amount of alcohol consumed, please answer the guideline for 20g of pure alcohol as follows: beer 500㎖ / sake 1 g / shochu 0.6 g / whisky 60㎖ / wine 1 glass / canned shochu 500㎖.

*As an example of calculating the amount of alcohol consumed, if 400㎖ of alcohol with an alcohol content of 4% is consumed, 400 × 0.04 = 16 g.

| 5-2．How much do you drink per day? | ・Not drinking at all  ・Less than 20ｇ  ・20–40ｇ  ・40–60ｇ  ・60–80ｇ  ・More than 80 g |
| --- | --- |

**6．Menstruation**

| 6-1．Are you currently menstruating? | ・Yes  ・No |
| --- | --- |

For those who selected「Yes」

| 6-2．Do you currently have irregular menstruation? | ・Yes  ・No |
| --- | --- |
| 6-3．Have you had periods of amenorrhoea or rare menstrual periods in the past?  ＊Except before and after childbirth. | 1．No  2．6–12 years old  3．13–15 years old  4．16–18 years old  5．19–22 years old  6．23–25 years old  7．26–30 years old  8．31–35 years old  9．36–39 years old  10．After 40 years old |

For those who selected「No」

| 6-2．When did you reach menopause? | 1．39 years old  2．40 years old  3．41 years old  4．42 years old  5．43 years old  6．44 years old  7．45 years old  8．46 years old  9．47 years old  10．48 years old  11．49 years old  12．After 50 years old |
| --- | --- |
| 6-3．Have you had irregular menstrual periods in the past? | ・Yes  ・No |
| 6-4．Have you had periods of amenorrhoea or rare menstrual periods in the past?  ＊Except before and after childbirth | 1．No  2．6–12 years old  3．13–15 years old  4．16–18 years old  5．19–22 years old  6．23–25 years old  7．26–30 years old  8．31–35 years old  9．36–39 years old  10．After 40 years old |

**7．Childbirth**

| 7-1．Have you ever given birth? | ・No  ・１ time  ・２ times  ・３ times  ・４ times |
| --- | --- |
| 7-2. How old were you when you had your first child? | ・20–25 years old  ・26–29 years old  ・30–35 years old  ・36–39 years old  ・After 40 years old  ・Others |
| 7-2. How old were you when you had your second child? | ・20–25 years old  ・26–29 years old  ・30–35 years old  ・36–39 years old  ・After 40 years old  ・Others |
| 7-2. How old were you when you had your third child? | ・20–25 years old  ・26–29 years old  ・30–35 years old  ・36–39 years old  ・After 40 years old  ・Others |
| 7-2. How old were you when you had your fourth child? | ・20–25 years old  ・26–29 years old  ・30–35 years old  ・36–39 years old  ・After 40 years old  ・Others |
